# Inter-organellar and systemic responses to impaired mitochondrial matrix protein import in skeletal muscle

**DOI:** 10.1038/s42003-022-04034-z

**Published:** 2022-10-05

**Authors:** Nirajan Neupane, Jayasimman Rajendran, Jouni Kvist, Sandra Harjuhaahto, Bowen Hu, Veijo Kinnunen, Yang Yang, Anni I. Nieminen, Henna Tyynismaa

**Affiliations:** 1 Stem Cells and Metabolism Research Program, Faculty of Medicine, University of Helsinki, Helsinki, Finland; 2 Metabolomics Unit, Institute for Molecular Medicine Finland (FIMM), HiLIFE, University of Helsinki, Helsinki, Finland

**Keywords:** Metabolism, Mitochondria

## Abstract

Effective protein import from cytosol is critical for mitochondrial functions and metabolic regulation. We describe here the mammalian muscle-specific and systemic consequences to disrupted mitochondrial matrix protein import by targeted deletion of the mitochondrial HSP70 co-chaperone GRPEL1. Muscle-specific loss of GRPEL1 caused rapid muscle atrophy, accompanied by shut down of oxidative phosphorylation and mitochondrial fatty acid oxidation, and excessive triggering of proteotoxic stress responses. Transcriptome analysis identified new responders to mitochondrial protein import toxicity, such as the neurological disease-linked intermembrane space protein CHCHD10. Besides communication with ER and nucleus, we identified crosstalk of distressed mitochondria with peroxisomes, in particular the induction of peroxisomal Acyl-CoA oxidase 2 (ACOX2), which we propose as an ATF4-regulated peroxisomal marker of integrated stress response. Metabolic profiling indicated fatty acid enrichment in muscle, a shift in TCA cycle intermediates in serum and muscle, and dysregulated bile acids. Our results demonstrate the fundamental importance of GRPEL1 and provide a robust model for detecting mammalian inter-organellar and systemic responses to impaired mitochondrial matrix protein import and folding.

## Abstract (web summary)

Muscular pathologies, including muscular dystrophy, are caused by a number of cellular and metabolic perturbations; here, consequences of GRPEL1-null disrupted mitochondrial matrix protein import on muscle atrophy, metabolism, and stress responses are examined.

## Introduction

Mitochondria are key organelles in the regulation of cellular metabolism. The vital functions of mitochondria are largely performed by proteins imported into the organelle from cytosol. Nuclear-encoded mitochondrial proteome (~1500 proteins) is synthesized by cytosolic ribosomes and imported into mitochondria as precursor proteins^1^. The protein import processes utilize targeting signals and specialized import pathways depending on which mitochondrial compartment the protein is to be targeted^2^. Proteins directed to the mitochondrial matrix enter through the double membrane via the translocases of the outer membrane (TOM) and inner membrane (TIM23). Finally, the presequence translocase-associated motor (PAM) pulls the incoming proteins into the matrix. PAM has the mitochondrial heat shock protein 70 (mtHSP70) at its core, and its ATPase cycle for substrate protein binding and release is regulated by nucleotide exchange factors (NEFs) and J-domain proteins. NEFs facilitate the ADP/ATP exchange and substrate release. In mammalian mitochondria, two bacterial GrpE-like NEFs, GRPEL1 and GRPEL2, have been identified^3^, but the functional significance of the two is not clear. Both can regulate mtHSP70 function and form hetero-oligomeric complexes in vitro^4^. However, human variation data and studies in cultured cells have indicated an essential function only for GRPEL1, whereas GRPEL2 may have a stress-regulated role^5^. Furthermore, GRPEL1, but not GRPEL2, is able to complement Mge1, the sole mitochondrial NEF in *Saccharomyces cerevisiae*^4^.

Inability to import proteins into mitochondria results in cytosolic accumulation of mistargeted proteins, which activate proteotoxic stress response pathways^6,7^. Studies in yeast have supported a protective role for the stress responses to counteract the impaired mitochondrial protein import by inhibiting global protein synthesis and activating the proteasome^6,7^. Tissue-specific and systemic effects of compromised protein import in mammals have been studied less, however, other models of mitochondrial dysfunction have demonstrated that integrated stress response (ISR) is a common adaptation to mitochondrial defects^8–11^. The core event of ISR is the phosphorylation of eIF2a, which leads to a decrease in global protein synthesis and the induction of selected genes that together promote cellular recovery. However, severe stress can drive ISR signaling toward cell death^12,13^. ISR activated by mitochondrial dysfunction in mammals induces a transcriptional induction of secreted cytokines FGF21 and GDF15, and remodeling of one-carbon metabolism^9,11^, and it can be regulated by mTORC in skeletal muscle^14^. The transcriptional changes are driven by transcription factors ATF4, which is also the effector of the PERK arm of endoplasmic reticulum (ER) stress, or ATF5, depending on context^11,15,16^.

Here we show that GRPEL1 is essential from early development in mice, supporting its role as the main NEF for mtHSP70. GRPEL1 is critical for mitochondrial maintenance also in developed tissues, as we show that induced loss of GRPEL1 in skeletal muscle led to rapid muscle atrophy and shortened life span. We describe the robust mitochondrial, inter-organellar and systemic responses to GRPEL1 loss, providing a resource for understanding the consequences of mitochondrial protein import toxicity in mammals. Finally, we identify ACOX2 as an ATF4-regulated peroxisomal marker of ISR.

## Results and discussion

### Loss of mitochondrial co-chaperone GRPEL1 in skeletal muscle of mice causes rapid muscular atrophy

**Fig. 1:**
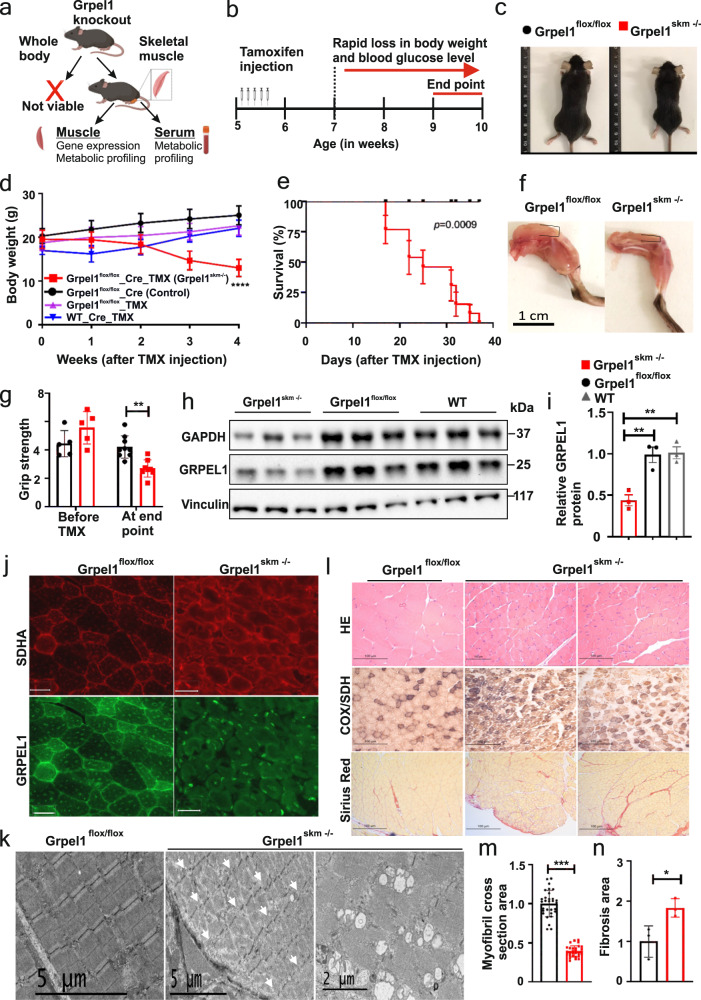
Inducible loss of mitochondrial co-chaperone Grpel1 in skeletal muscle of mice causes rapid muscular atrophy, retarded growth and shortened lifespan. **a** A schematic for the study of whole-body and skeletal muscle-specific knockouts of *Grpel1* (Created with BioRender.com). **b** Timeline of Cre-activation in mice by tamoxifen (TMX) injections. **c** Representative images of *Grpel1*^*skm*−/−^ (right) 4 weeks after tamoxifen injection and the control littermate *Grpel1*^*flox/flox*^ (left). **d** Body weight curve of male mice, starting from TMX injection. Four different groups of mice are shown: *Grpel1*^*skm*−/−^ are the inducible skeletal muscle-specific *Grpel1* knockout mice, *Grpel1*^*flox/flox*^_Cre are the floxed Cre mice without tamoxifen, *Grpel1*^*flox/flox*^_TMX are the floxed mice without Cre but with tamoxifen, and WT_Cre_TMX are wild type Cre mice with tamoxifen. (*n* = 10–12 per genotype). Tukey’s multiple comparisons test. ^∗∗∗∗^*p* ≤ 0.0001. **e** Survival curve of *Grpel1*^*skm*−/−^ mice analysed with Gehan-Breslow-Wilcoxon test. (*n* = 13 per genotype). **f** Representative images of quadriceps of *Grpel1*^*skm*−/−^ (right) and control littermate *Grpel1*^*flox/flox*^ (left) at sacrification point. **g** Grip strength of mice immediately before tamoxifen injection and at end point (*n* = 5–7 per genotype). **h** Immunoblot showing reduced GRPEL1 level in skeletal muscle of *Grpel1*^*skm*−/−^, in comparison to *Grpel1*^*flox/flox*^ and wild type (WT) mice. Vinculin is shown as loading control. **i** Quantification of GRPEL1 protein level in *Grpel1*^*skm*−/−^ muscle from immunoblot against Vinculin (*n* = 3 per genotype). **j** Representative immunohistochemistry images of quadriceps femoris (QF) from control and knockout mice with GRPEL1 and SDHA antibodies. Scale bar is 100 µm. **k** Representative electron microscopic images of QF muscle from control and knockout mice, showing the uneven width of sarcomere and discontinuous Z-line in knockout (left, arrows) and large vacuole structures identified in some fibers (right). **l** Representative Hematoxylin/Eosin, COX/SDH, and Sirius Red (fibrosis) staining images of QF muscle. **m** Quantification of cross-sectional area of myofibril in QF muscle (*n* = 30 representative myofibrils from 3 mice per genotype). **n** Quantification of fibrotic area of QF muscle (*n* = 3 mice per genotype).

To clarify the in vivo role of GRPEL1 in mammalian mitochondria, we intended to generate whole-body *Grpel1* knockout mice. Heterozygous mice (*Grpel1*^*+/−*^) were viable with no apparent histological abnormalities (Supplementary Fig. 1a–d). However, F1 heterozygous crosses provided no homozygous *Grpel1*^−/−^ pups, which were also lacking at embryonic day E8.5, indicating early developmental lethality (Fig. 1a and Supplementary Fig. 1e). These results confirm that GRPEL1 is essential in mammals.

To study tissue-specific consequences of GRPEL1 loss, we generated inducible skeletal muscle-specific *Grpel1* knockout mice (*Grpel1*^*skm*−/−^) using tamoxifen (TMX)-inducible Cre recombinase and human α-skeletal actin (HAS) promotor (*HSA-Cre* mice) (Supplementary Fig. 2a). TMX-inductions were started for the mice at the age of five weeks. Unexpectedly, already within two weeks from the induction, *Grpel1*^*skm*−/−^ mice started to lose weight rapidly unlike untreated littermates (*Grpel1*^*flox/flox*^) or TMX-treated wild type mice (Fig. 1b–d and Supplementary Fig. 2b, c). Indirect calorimetry, performed after two weeks of tamoxifen treatment, showed no significant differences in movement, oxygen consumption, respiratory exchange ratio or heat production between *Grpel1*^*skm*−/−^ mice and controls (Supplementary Fig. 2d, e). Moreover, glucose tolerance and insulin sensitivity were not significantly altered in *Grpel1*^*skm*−/−^ mice (Supplementary Fig. 3a, b). However, the phenotype advanced to the sacrification point (30% weight loss, kyphosis, reduced movement) within five weeks from TMX-induction (Fig. 1e). At the end point, blood glucose was significantly reduced (Supplementary Fig. 3c). Skeletal muscles appeared atrophic, and grip strength was reduced (Fig. 1f, g). Detected by immunoblotting, the GRPEL1 protein level in quadriceps femoris (QF) muscle was reduced to about 40% of control level (Fig. 1h, i). Immunostaining of the QF muscle with GRPEL1 antibody further indicated reduced GRPEL1 protein in *Grpel1*^*skm*−/−^ mice (Fig. 1j). Electron micrographs of QF showed an uneven width of sarcomeres and discontinuous Z-lines (Fig. 1k), and large mitolysosome-like structures^17^ in some fibres (Fig. 1k). Histological staining of QF with Hematoxylin & Eosin (HE) and Sirius Red in *Grpel1*^*skm*−/−^ muscle revealed shrinkage of myofibrils, and fibrosis, respectively (Fig. 1l–n). Histochemical activity staining of cytochrome c oxidase (COX) and succinate dehydrogenase (SDH) showed some COX-negative muscle fibers, but none of those were SDH-positive ragged red fibers (Fig. 1l). These results indicated that the loss of GRPEL1 caused rapid atrophy and disorganization of skeletal muscle.

### Loss of GRPEL1 leads to transcriptional repression of mitochondrial metabolic pathways

**Fig. 2:**
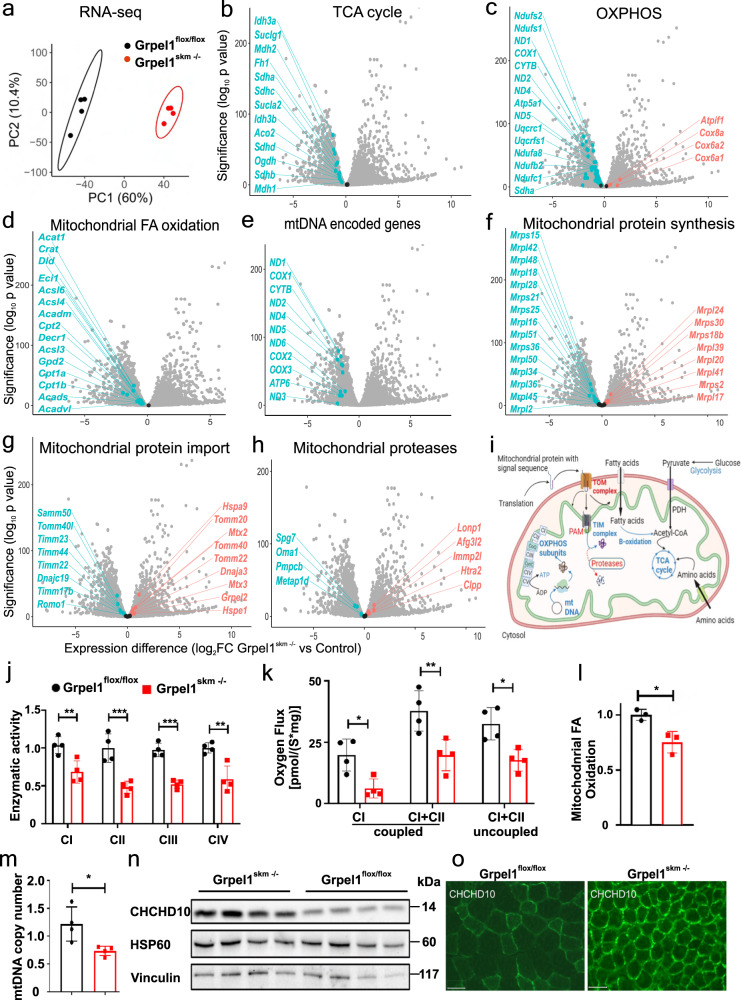
Intramitochondrial response to Grpel1 loss in skeletal muscle. **a** Principal component analysis (PCA) for RNA sequencing data from QF of *Grpel1*^*skm*−/−^ and control mice (*n* = 4 per genotype). **b**–**h** Volcano plots showing the changes in gene expression in different mitochondrial pathways. **i** Graphical representation of the altered mitochondrial pathways in *Grpel1*^skm−/−^ muscle. Red color (arrows and text) indicates upregulation whereas the blue color (arrows and text) indicates downregulation (Created with BioRender.com). **j** Enzymatic activity of mitochondrial respiratory chain complexes, in mitochondria isolated from QF muscle, and normalized to control (*n* = 4 per genotype). **k** CI‐coupled, CI + CII‐coupled and uncoupled (with FCCP) respiration in QF muscle homogenates (*n* = 4 per genotype). **l** Quantification of fatty acid oxidation capacity of mitochondria, isolated from QF muscle, and normalized to control (*n* = 3 per genotype). **m** Quantification of mtDNA copy number in QF muscle of *Grpel1*^*skm*−/−^ and control mice (*n* = 4 per genotype). **n** Immunoblot of total protein lysates from QF muscle with CHCHD10 and HSP60 antibodies. **o** Representative images of immunohistochemistry staining of QF muscle with CHCHD10 antibody. Scale bar is 100 µm.

Next, we analyzed the transcriptome of *Grpel1*^*skm*−/−^ QF by RNA sequencing to identify transcriptional responses to GRPEL1 loss in muscle. Principal component analysis (PCA) indicated that the muscle of *Grpel1*^*skm*−/−^ mice greatly differed from control muscle (Fig. 2a), with more than 6000 differentially expressed genes (FDR < 0.01) (Supplementary Data 1). These included transcriptional repression of the major mitochondrial metabolic pathways such as the TCA cycle, OXPHOS (RC-complex subunits), and mitochondrial fatty acid (FA) oxidation (Fig. 2b–d; Supplementary Data 2, 3; Supplementary Fig. 4). Mitochondrial DNA encoded transcripts were reduced (Fig. 2e), as well as mtDNA copy number (Fig. 2m). Most transcripts for mitochondrial ribosome subunits were downregulated (Fig. 2f). The shutdown of the key metabolic functions was supported by reduced respiration, as determined by respirometry, with lower OXPHOS complex I-IV activities (Fig. 2j, k), and reduced FA oxidation in mitochondria isolated from *Grpel1*^*skm*−/−^ muscle (Fig. 2l). As an exception to the downregulation of OXPHOS transcripts, tissue-specific isoforms of COX subunits *Cox6a1*, *Cox6a2* and *Cox8a*^18^ were increased. Similarly, *Atpif1*, which codes an inhibitor protein of ATP synthase (IF_1_) with the ability to conserve ATP at the expense of membrane potential when mitochondrial respiration is inhibited^19^, was increased (Fig. 2c). Interestingly, a few mitochondrial pathways were also transcriptionally upregulated in *Grpel1*^*skm*−/−^ muscle (Supplementary Data 3). Among those were some TOM (*Tomm34, 22, 40*, and *20*), but not TIM subunits (Fig. 2g). PAM complex transcripts *Hspa9* (mtHSP70) and *Grpel2*, the paralog of *Grpel1*, were also increased as well as mitochondrial chaperones *Dnaja3* (HSP40) and *Hspe1* (HSP10). The expression of some mitochondrial proteases, such as matrix proteases *Clpp* and *Lonp1*, and inner membrane or intermembrane space (IMS) proteases *Afg3l2*, *Immp2l* and *Htra2*, was increased (Fig. 2h). These findings may suggest that GRPEL1 loss activates mitochondrial chaperones and proteases to balance impaired protein folding within mitochondria, although this cannot be concluded from transcriptional data. We also observed that the expression of amyotrophic lateral sclerosis (ALS)-linked gene^20^ *Chchd10* was significantly increased in *Grpel1*^*skm*−/−^ muscle, which we also detected on protein level by Western blotting and immunohistochemistry (Fig. 2n, o). The exact function of the mitochondrial IMS protein CHCHD10 is unknown, but it has been proposed to suppress protein aggregate (TDP-43) formation in mitochondria^21^. The highly induced expression of *Chchd10* in *Grpel1*^*skm*−/−^ muscle, in which the majority of the altered mitochondrial transcripts were downregulated (465 MitoCarta3.0^22^ genes downregulated, 183 upregulated, Supplementary Data 3), suggests that it may have a role in mitochondrial protein import stress. In summary, these transcriptome findings indicated the shutdown of mitochondrial metabolic functions as a result of GRPEL1 loss, with induction of compensatory pathways contributing to mitochondrial proteostasis (Fig. 2i).

### Transcriptional alterations indicative of inter-organellar communication in Grpel1 knockout muscle

**Fig. 3:**
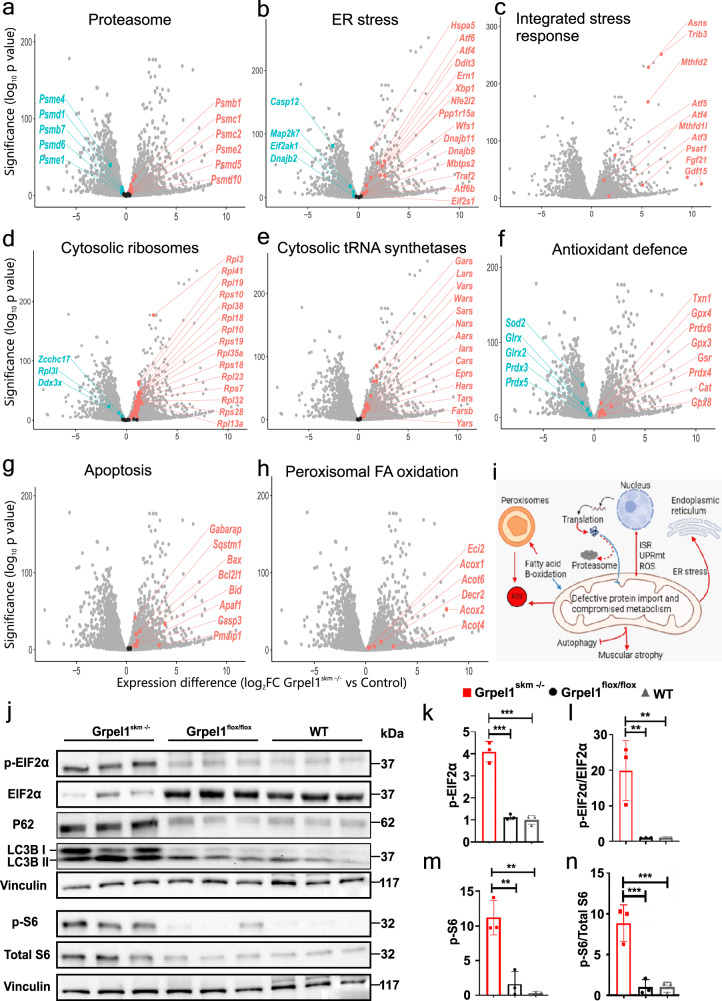
Organelle crosstalk in response to Grpel1 loss in skeletal muscle. **a**–**h** Volcano plots for different pathways indicating changes in gene expression due to loss of *Grpel1* in skeletal muscle. **i** Graphical representation of crosstalk between dysfunctional mitochondrial, due to loss of *Grpel1*, with other cellular organelles. Red color (arrows) indicates upregulation whereas the blue color (arrows) indicates downregulation (Created with BioRender.com). **j** Immunoblot of total protein lysates from QF muscle of *Grpel1*^*skm*−/−^, *Grpel1*^*flox/flox*^ and WT mice. **k**–**n** Quantification of p-EIF2α level, p-EIF2α/total EIF2α ratio, p-S6 level and p-S6/total S6 ratio in skeletal muscle of *Grpel1*^skm−/−^, *Grpel1*^*flox/flox*^ and WT mice. Vinculin was used as the loading control (*n* = 3 per genotype).

We then focused on transcriptome alterations affecting pathways outside mitochondria to address inter-organellar communication as a response to impaired mitochondrial protein import. The expression of some, but not all, proteasome subunits were increased (Fig. 3a). ER stress and ISR were among the most significantly induced pathways, supporting that GRPEL1 loss was leading to mistargeted proteins and proteotoxic stress (Fig. 3b, c). Secreted ISR cytokine genes *Fgf21* and *Gdf15*, which have virtually no expression in healthy skeletal muscle, were among the most highly induced transcripts, along with other stress response genes such as *Ddit3* (CHOP), *Mthfd2* and *Psat1*. These stress responses were accompanied by increased eIF2α phosphorylation (Fig. 3j–l), and increased expression levels of *Atf4* (2.3-fold), *Atf5* (5.0-fold) and *Atf6* (3.8-fold), the latter being the effector of ATF6 arm of ER stress^23^. We also observed a striking elevation in transcripts of cytosolic ribosome subunits and cytosolic tRNA synthetases (Fig. 3d, e). This contrasts with attenuation of protein synthesis in acute proteotoxic stress and suggests that the *Grpel1*^*skm*−/−^ muscle was in prolonged stress leading to enhanced protein synthesis^13,24–26^. Indeed, phosphorylation of ribosomal protein S6 (p-S6), a downstream target of mTORC, was increased in *Grpel1*^*skm*−/−^ muscle (Fig. 3j, m, n). Stress-induced increase in protein synthesis has been shown to lead to oxidative stress and cell death^25^. In *Grpel1*^*skm*−/−^ muscle many antioxidant defense genes such as *Cat* (catalase) were upregulated, but mitochondrial *Sod2*, *Prdx3*, *Prdx5*, and glutaredoxins were downregulated (Fig. 3f). Transcripts related to autophagy and apoptosis were also increased (Fig. 3g). Recent study has shown that ISR-linked pathology in mitochondrial myopathy is associated with stalled autophagy in the advanced stage when mTORC becomes activated^17^. Accordingly, we observed a clear increase in p62/SQSTM1 levels, and a shift in LC3BI/II ratio in *Grpel1*^*skm*−/−^ muscle by Western blot (Fig. 3j).

### ACOX2 is a peroxisomal ISR marker

**Fig. 4:**
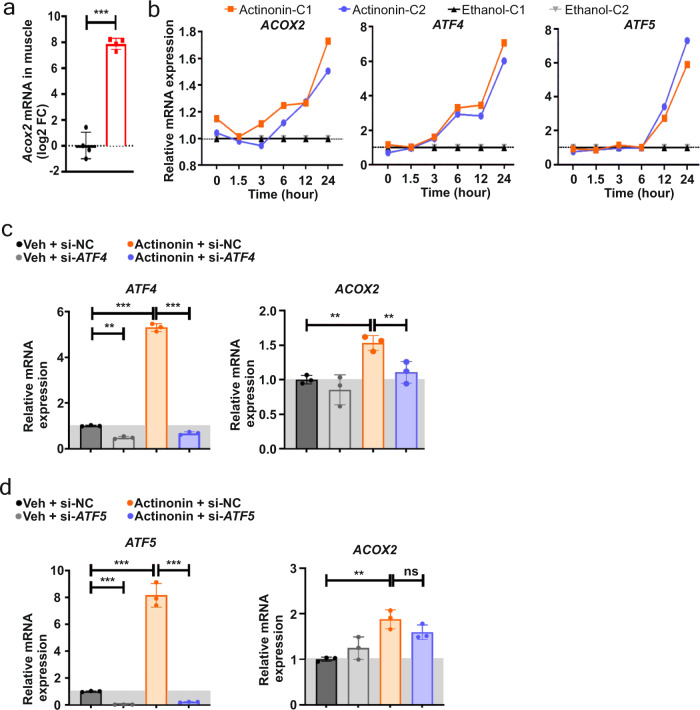
ACOX2 is a peroxisomal marker for ISR. **a** Quantification of *Acox2* mRNA expression in QF muscle of Grpel1^skm −/−^ mice (*n* = 4 per genotype). **b** Timeline showing the induction of *ACOX2*, *ATF4* and *ATF5* mRNA expression in human fibroblasts (C1 and C2 are independent control fibroblast lines) after treatment with actinonin, compared with vehicle (ethanol). **c** Quantification of *ATF4* and *ACOX2* mRNA expression after siRNA-mediated knockdown of ATF4 followed by 24 h of actinonin treatment (*n* = 3 per treatment). **d** Quantification of *ATF5* and *ACOX2* mRNA expression after siRNA-mediated knockdown of ATF5 followed by 24 h of actinonin treatment (*n* = 3 per treatment).

Interestingly, we also identified increased gene expression on the peroxisomal beta-oxidation pathway (Fig. 3h). In particular, the peroxisomal Acyl-CoA oxidase 2 (*Acox2*) showed high induction (Figs. 3h and 4a), suggesting that peroxisomal metabolism may be activated in muscle in response to mitochondrial shutdown. ACOX2 is involved in the beta-oxidation of bile acid intermediates, resulting in the CoA esters of cholic and chenodeoxycholic acid^27^. Reminiscent of key ISR markers such as *Fgf21* and *Gdf15*, *Acox2* is barely expressed in normal skeletal muscle, but had high expression in *Grpel1*^*skm*−/−^ muscle. *Acox2* expression has not been previously linked to ISR, but we noticed that it was also highly expressed in the *Dars2* knockout mice, which have highly induced ISR owing to impaired mitochondrial translation^13^. According to ChIP-seq data, ATF4 may regulate the expression of *Acox2*^25^. We thus suspected that *Acox2* could be a peroxisomal marker of ISR. To test whether its elevation was specific to mouse muscle, we studied *ACOX2* expression in human skin fibroblasts treated with actinonin, a known ISR inducer that blocks mitochondrial protein synthesis^28^ and induces *ATF4* and *ATF5* expression. Intriguingly, actinonin treatment induced *ACOX2* expression (Fig. 4b), supporting that it has a yet uncharacterized role in proteotoxic stress. Next, we tested if *ACOX2* induction was regulated by ATF4 or ATF5 using siRNA mediated knockdown of these transcription factors. The results showed that actinonin-induced expression of *ACOX2* was repressed by *ATF4* siRNA but not by *ATF5* siRNA (Fig. 4c, d), demonstrating that *ACOX2* is an ATF4-regulated ISR target gene. Taken together, these results indicate that GRPEL1 loss in skeletal muscle activates crosstalk with the nucleus, ER, proteasome and the peroxisomes. However, the activation of the stress responses does not rescue from pro-apoptotic signaling and muscle atrophy (Fig. 3i).

### GRPEL1 loss in skeletal muscle leads to systemic metabolic alterations

**Fig. 5:**
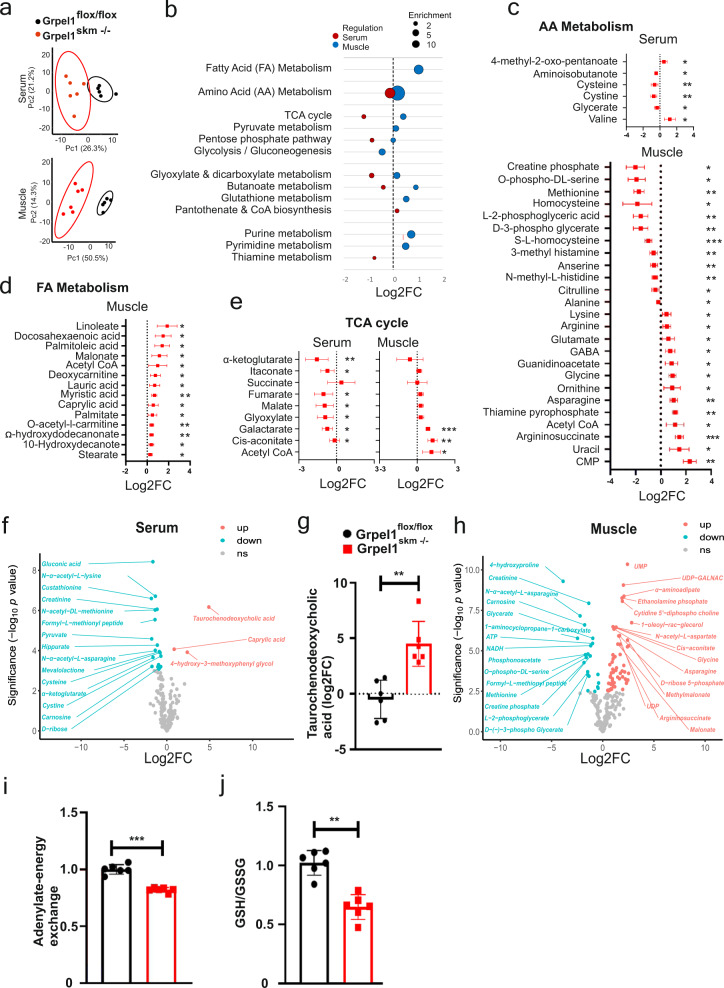
Metabolite profiling of QF muscle and serum of *Grpel1*^skm−/−^mice. **a** PCA plots for metabolite profiling data from serum and QF muscle. The ellipses show 95% confidence intervals (*n* = 6 per genotype). **b** Pathway enrichment analysis of altered metabolites in muscle and serum. Mean fold change of significantly altered metabolites is considered for each pathway. The size of the circle indicates the gene ratio, and color indicates the sample source (red-serum, blue-muscle). **c** Altered metabolites of amino acid (AA) metabolism in *Grpel1*^skm −/−^serum and muscle. **d** Altered metabolites of fatty acid (FA) metabolism in *Grpel1*^skm −/−^muscle. **e** Altered metabolites of TCA cycle in *Grpel1*^skm −/−^ serum and muscle. **f** Volcano plot indicating significantly altered (*p*-value < 0.05) metabolites in *Grpel1*^skm−/−^serum. **g** Quantification of Taurochenodeoxycholic Acid in serum. **h** Volcano plot indicating significantly altered metabolites in *Grpel1*^skm −/−^muscle. **i** The adenylate-energy charge calculated according to^29^ in *Grpel1*^skm−/−^ muscle relative to control. **j** Reduced and oxidized glutathione ratio in *Grpel1*^skm −/−^ muscle relative to control.

Finally, we performed a targeted metabolite analysis of QF and serum to identify tissue-specific and systemic consequences of *Grpel1* loss (Supplementary Data 4). The metabolite profile of *Grpel1*^*skm*−/−^ was substantially different from that of the control mice, both in serum and muscle, as indicated in the PCA plot (Fig. 5a). Most altered metabolites in serum were decreased, whereas in muscle both increases and decreases in metabolite levels were identified (Fig. 5f, h). The results indicated that glycolysis was decreased, and fatty acids (FA, unsaturated FA, and ketone bodies) increased in muscle, suggesting a metabolic shift (Fig. 5b, c, d). A combined analysis revealed a possible metabolite exchange between serum and muscle (Fig. 5b), as some TCA cycle metabolites appeared depleted in serum and increased in muscle (Fig. 5e), although any exchange cannot be concluded without more detailed studies. Altered amino acid metabolism indicated protein breakdown in muscle (Fig. 5c). Creatinine and carnosine were depleted in both serum and muscle, consistent with reduced muscle mass (Fig. 5f, h). Elevation in nucleotide metabolism was detected in muscle (Fig. 5c, h), as previously reported in ISR-linked mitochondrial myopathy^9^. Adenylate–energy charge ratio^29^ in muscle was lower (Fig. 5i), indicating poor energy status. Lower ratio of reduced (GSH) to oxidized (GSSG) glutathione in muscle reflected higher oxidative stress (Fig. 5j). The most increased serum metabolite was taurochenodeoxycholic acid (TCDCA) (Fig. 5f, g), a taurine-conjugated bile acid, which was also reported increased in patients with mitochondrial myopathy^9^. Thus, our results accentuate the link between mitochondrial impairment in skeletal muscle and systemic bile acid dysregulation.

## Conclusions

This study shows that GRPEL1 is an essential NEF in mammalian mitochondria and cannot be compensated by GRPEL2. The loss of GRPEL1 induces a robust proteotoxic stress response in the cytosol and also within mitochondrial matrix. These effects can be accounted to inhibited protein import into mitochondria and compromised protein folding by mtHSP70, which leads to accumulation of misfolded and mistargeted proteins, prone to aggregation. The muscle atrophy caused by GRPEL1 loss is very rapid, and thus the associated responses are also pronounced. Therefore, these mice offer a clear-cut model for elucidating the muscle-specific and systemic responses to impaired mitochondrial protein import and folding. We highlight a potential role for Chchd10 in mitochondrial protein import stress, and identify Acox2 as a peroxisomal marker of ISR. This study emphasized the crosstalk between mitochondria and peroxisomes in demand of a metabolic shift when mitochondrial functions become inhibited. The identified survival responses do not rescue the severe phenotype of Grpel1^skm−/−^ mice but may be beneficial in understanding and treating milder muscle pathologies.

## Methods

### Mouse maintenance

Animal experiments were performed in compliance with the national ethical guidelines set by the European Union and were approved by the National Animal Experiment Board (Project Authorisation Board). The ethical practice of handling laboratory animals was strictly followed throughout the procedures. The mice were housed at 22 °C with 12 h light/dark cycles with *ad libitum* access to standard rodent food and water.

### Knockout mouse generation

Embryonic stem cells (ES) with KO first allele targeted for *Grpel1* were obtained from European Mouse Mutant Cell Repository (EuMMCR). ES cells were expanded and then injected into the blastocyst of C57BL/6NCrl mice in Biocenter Oulu Transgenic Core Facility. The chimeras were genotyped and bred to generate the *Grpel1* heterozygotes (*Grpel1*^+/-^), which were then intercrossed in attempt to generate the whole-body homozygous knockout of *Grpel1* (*Grpel1*^−/−^). To remove the Neo cassette, *Grpel1*^+/-^ mice were crossed with transgenic mice expressing Flp recombinase. The mice without Neo cassette were then bred with HSA-Cre-ERT2 mice, which express tamoxifen-inducible Cre-ERT2 recombinase under the control of Human skeletal muscle α-actin gene (HSA)^30^. Tamoxifen (Sigma: T5648) was dissolved in corn oil (Sigma: C8267) (20 mg/ml) by gently shaking at 37 °C overnight and stored at −20 °C. For injection, 5-week-old mice were given 100 µl of tamoxifen for 5 consecutive days with a 22 g syringe, intraperitoneally (IP). The mice were then kept in quarantine for 3 days. After quarantine, the mice were transferred to new cages and monitored. Body weights were recorded before and after the treatment every week.

### Whole-body metabolism and body composition

Oxymax Comprehensive Lab Animal Monitoring System (CLAMS; Columbus Instruments) was used to measure the oxygen consumption, carbon dioxide production and spontaneous activity of the mice. The mice were kept in individual cages that were regulated for temperature ( + 22 °C) and parameters were recorded for 24 h, following an overnight adaptation before recordings. The body composition, i.e., fat and lean mass, of the mice was estimated with Bruker minispec LF50 Body Composition Analyzer.

### Grip strength

Muscle strength was determined with a grip strength meter. The mouse was placed over the grid, gently pulled by its tail, and allowed to grasp the mesh grid by its fore and hind limbs. Six consecutive measurements were performed with a minute interval for each mouse. The mean maximal grip strength was recorded and then normalized to the body weight of individual mouse.

### Glucose and insulin tolerance tests

A 6 h fasting period was set for the glucose tolerance test by removing food from the cages. The basal blood glucose was measured by tail vein puncture and then the mice were given glucose (1 g/kg of body weight) by IP injection. The blood glucose levels were measured at 15, 30, 60, 90, and 120 min with a glucometer (Accu-Chek Aviva Glucose Meter, Roche). For the insulin sensitivity test, basal blood glucose level was measured by puncturing the tail vein. Then the mice were given intraperitoneal insulin injection (0.8 U/kg of body weight) (Novolin R, Novo Nordisk) and the blood glucose was measured at 15, 30, 60, 90, and 120 min after the injection.

### Histology

After the tissue samples were collected, they were fixed in 10% buffered formalin for one to three days at 4 °C. Tissues were then processed and paraffinized using Sakura Tissue-Tek VIP processor and next day embedded in paraffin blocks. For Hematoxylin and Eosin (HE) staining^14,31^, the tissue blocks were sectioned and stained with Hematoxylin for 1 min and with Eosin for 2 min, with washes in between the stains. Dehydration was done with absolute EtOH, cleared with xylene. The sections were then mounted with Pertex mounting medium (Histolab 00811). For the analysis of collagenization in skeletal muscle, paraffin samples were stained with Picrosirius Red staining. Samples were first de-paraffinized with xylene and rehydrated using descending EtOH series. After rehydration, samples were stained with Weigert´s haematoxylin for 8 min to stain nuclei and then stained with 0.1% Sirius Red (Sigma, 365548) in 1.3% picric acid for one hour. Samples were then washed twice with acidified water (5 ml acetic acid in one liter of distilled water), dehydrated with ascending EtOH series, cleared with xylene and mounted with Pertex mounting medium (Histolab 00811). For the stainings of cytochrome c oxidase (COX) and succinate dehydrogenase (SDH) activities^14,31^, the tissues were freshly embedded in small amount of OCT medium (Tissue-Tek) and frozen in 2-methylbutane bath in liquid nitrogen. After freezing tissues were embedded with bigger amount of OCT and kept in −20 °C. The sectioning was done using Leica CM3050S cryostat. The muscle sections were incubated for 30 min in COX and 40 min in SDH straight on the object glass and washed in between. Dehydration was done with ascending EtOH series, cleared with xylene and then mounted with Pertex mounting medium (Histolab 00811). All the stained sections were imaged by Axioplan 2 Universal Microscope (Zeiss).

### Immunohistochemistry

Immunohistochemistry was done both from formalin-fixed paraffin embedded samples and frozen samples. Formalin-fixed paraffin embedded samples were first deparaffinized and rehydrated using xylene and descending EtOH series. Antigen retrieval was done by heating the samples 15 min in pressure cooker at 95 °C in 10 mM EDTA or 0.1 M citrate buffer, pH 6. Frozen samples were first dried 20 min at room temperature and rinsed with 0.1% PBS-Triton-X. After this all steps were the same for both sample types. Nonspecific staining was blocked by incubating samples in blocking buffer (1.5% Normal goat serum  + 5% fetal bovine serum in 0.1% PBS-Triton X-100) for 1 h at room temperature. The primary antibodies were diluted in Dako antibody diluent (Agilent S3022) and incubated overnight at  + 4 °C. The following primary antibodies were used: anti-GRPEL1 (NBP1-54665, Novus Biologicals), mouse anti-SDHA (ab14715, Abcam), rabbit anti-CHCHD10 (HPA003440, Sigma). Next day samples were washed three times with 0.1% PBS-Triton X-100 and secondary antibodies were diluted in Dako antibody diluent and incubated 30 min at RT. The samples were then washed 3x with 0.1% PBS-Triton X-100 and mounted with Vectashield mounting media with DAPI (Vector laboratories H-1200). Images were taken with Leica DM5000B wide field microscope.

### Mitochondrial DNA copy number analysis

Total DNA was extracted from snap frozen QF muscle of control and *Grpel1*^*skm−/−*^ mice by phenol-chloroform extraction method and quantified using Spectrophotometer (DeNovix, DS-11 Series). 30 ng of isolated DNA was used as template. The mitochondrial DNA was quantified by amplifying the mitochondrial *12* *S* rRNA gene and normalized to nuclear *RBM15* gene. Primers used were: *12S*_Forward: AGGAGCCTGTTCTATAATCGATAAA, *12* *S*_Reverse: GATGGCGGTATATAGGCCGAA, *RBM15*_Forward: GGACAGTTTTCTT GGGCAAC and *RBM15*_Reverse: AGTTTGGCCCTGTGAGACAT.

### High-resolution respirometry

Total tissue lysates were used in Oroboros for respiration measurement. Tissue lysates in MiR05 (0.5 mM EGTA, 3 mM MgCl_2_, 60 mM K-lactobionate, 10 mM KH_2_PO_4_, 20 mM HEPES, and 110 mM sucrose, pH 7.1) were taken to the chamber. We measured the response in oxygen flux for CI-coupled respiration after adding malate (final concentration 2 mM), pyruvate (10 mM), glutamate (20 mM), ADP (5 mM), and cytochrome C subsequently into the chamber. The CI + CII coupled respiration was measured with the addition of succinate (10 mM). Subsequent addition of carbonylcyanide-4-(trifluoromethoxy)-phenylhydrazone (FCCP) (2 mM) provided maximal respiration (CI + CII uncoupled respiration) of mitochondria. Basal respiration was measured by inhibiting the reaction with rotenone (0.1 μM) and antimycin (2.5 μM). Total protein concentration of tissue lysate was used for normalization.

### Respiratory chain enzyme activity measurements

Isolated mitochondria were used for measuring RC complex activity. We isolated the mitochondria from skeletal muscle and followed the protocol of Spinazzi et al. 2012^32^ for the measurement of enzymatic activity of CI-CIII. Complex IV activity was measured using Oroboros from flux response to ascorbate (10 μM) and TMPD (2.5 μM) after inhibiting CIII with antimycin A. The complete reaction was compromised by adding Azide (50 μM) to the chamber. We normalized the enzyme activities to total protein concentrations.

### Immunoblotting

The tissue samples were snap frozen in liquid nitrogen and stored in −80 °C. About 20 mg of snap frozen tissues were taken and homogenized in RIPA buffer (Cell signaling technology) containing Halt™ Protease-inhibitor Cocktail (ThermoFisher) with Fast Prep w-24 Lysing Matrix D (MP Biomedical) and Precellys w-24 (Bertin Technologies). Protein concentrations were quantified with Bradford method (Bio-Rad). 20 μg of protein per sample were loaded into 10% stain free polyacrylamide gels (Bio-Rad) and transferred to nitocellulose membranes using transfer-blot turbo transfer system (Bio-Rad). The membranes were then blocked with 5% milk in 1X TBS-T to avoid nonspecific binding. The primary antibodies were prepared in 1% BSA/TBS-T with the concentrations recommended by the manufacturers. After subsequent washing, membranes were incubated with following primary antibodies overnight at + 4 °C: rabbit anti-GRPEL1 (NBP1-54665, Novus Biologicals), goat anti-HSP60 (sc-1052, Santa Cruz Biotechnology), rabbit anti-GAPDH (14C10, Cell Signaling), rabbit anti-CHCHD10 (HPA003440, Sigma), rabbit anti-EIF2α (ab26197, Abcam), rabbit anti-p-EIF2α (phospho S51) (ab32157, Abcam), rabbit anti-S6 ribosomal protein (2217, Cell signaling), rabbit anti-phospho-S6 ribosomal protein (Ser 240/244) (5364, Cell signaling), rabbit anti-LC3B (NB600-1384, Novus Biologicals), mouse anti-vinculin (V9264, Sigma) and mouse anti-p62 (2C11, Abnova). Membranes were washed and then incubated with respective HRP conjugated secondary antibodies for 1 h in room temperature. The Clarity Western ECL Substrate (BioRad) was used to develop protein band signals, detected by Chemidoc XRS + Molecular Imager (Bio-Rad). Quantification was done with the ImageLab software (Bio-Rad).

### Mitochondrial fatty acid oxidation measurement

The QF muscle samples (100 mg) were homogenized in STE buffer (0.25 M sucrose, 10 mM Tris, 1 mM EDTA) and mitochondria were isolated. The isolated mitochondrial fraction was incubated in a reaction buffer (14^C^-radiolabeled oleic acid (NEC317250UC- Perkin Elmer), 2 mM ATP, and 0.5 mM dithiothreitol) for 40 min at 37 °C. The CO_2_ released from fatty acid oxidation were trapped in 1 M NaOH-soaked filter paper and measured for acid-soluble metabolites using a scintillation counter.

### Transmission electron microscopy

The tissue samples were fixed in 2.5% glutaraldehyde for 2 h and stored in 2% PFA at 4 °C. Tissue samples were embedded into blocks, cut into thin sections and stained according to the standard procedure of the Electron Microscopy Unit of the Institute of Biotechnology, University of Helsinki. JEOL JEM-1400 Plus transmission electron microscope was used for imaging and ImageJ software for quantifications.

### RNA isolation and sequencing

Total RNA was isolated from snap frozen QF with miRNAeasy RNA extraction kit (Qiagen). The tissue samples were homogenized using Fast Prep w-24 Lysing Matrix D (MP Biomedical) and Precellys w-24 (Bertin Technologies). The NEBNext Ultra II Directional RNA Library Prep Kit for Illumina was used to generate sequencing libraries. From 1 µg of total RNAs, the polyadenylated mRNAs were captured with NEBNext Poly(A) mRNA Magnetic Isolation Module. The captured mRNAs were fragmented and then primed with random primers to generate the cDNA. The Agencourt AMPure XP beads were used to purify the double stranded cDNA. The end-prep reaction was performed using the end-prep reaction buffer and enzyme mix. The final PCR enrichment was carried after the adaptor ligated DNA went through a bead-based size selection. The amplified library was then purified using AMPure XP Beads and its quality was assessed by TapeStation (Agilent High Sensitivity D5000 assay) and library quantity by Qubit. The prepared libraries were then sequenced with Illumina NextSeq500-system (Mid Output 2 × 75 bp).

Raw data (bcl-files) was demultiplexed with bcl2fastq2 (v2.20.0.422; Illumina), removing adapter sequences and excluding reading reads that were too short (< 35 bp). Additional trimming for remaining adapter sequences, ambiguous (N) and low-quality bases (Phred score < 25) was done using cutadapt^33^. We also excluded read pairs that were too short (< 25 bp) after trimming. The filtered read pairs were mapped to the mouse reference genome (GRCm38) with STAR (v. 2.5.4b)^34^. Gene expression was analysed with R (v.4.1.1)^35^. Read pairs mapping to exons were used to extract gene expression counts using Rsubreads (v.2.6.4)^36^. Duplicates, chimeric and multimapping reads were excluded, as well as reads with low mapping score (MAPQ < 10). The read count data was analyzed with DESeq2 (v.1.32.0)^37^, comparing knockout to wild type. Genes with low read depth (< 50 reads in total) were excluded.

The volcano plots were generated with ggplot2 (v.3.3.5)^38^ using the fold change and p values obtained from DESeq2. PCA was calculated with prcomp using log1p-transformed normalized counts. The differentially expressed genes (FDR < 0.01) were analyzed for enrichment separately for the up- and down-regulated genes using clusterProfiler^39^ against the Reactome pathways^40^, KEGG^41^, Gene ontology^42^ and WikiPathways^43^.

### Actinonin treatment

Human skin fibroblasts obtained from two healthy volunteers were used for the experiments (C1 and C2). Briefly, cells were cultured at 37°C, in a humidified atmosphere, normoxia and 5% CO_2_. Fibroblasts were maintained in DMEM media (Lonza #12-614 F) supplemented with 2mM L-glutamate (Life Technologies #250300081), 10% FBS (Life Technologies #10270106), 1x penicillin/streptomycin (Life Technologies #15140122) and 50 µg/ml uridine (Sigma #U3003). Once confluent, fibroblasts were treated with 150 μM actinonin (Sigma, A6671) for 24 h and samples were collected at 0 h, 1.5 h, 3 h, 6 h, 12 h and 24 h. Ethanol was used as vehicle for actinonin. After treatment, fibroblasts were manually collected by scraping on ice and samples were stored at −80 °C until further processing. Total RNA from fibroblast cultures was isolated using NucleoSpin RNA extraction kit (Macherey-Nagel). RNA was reverse transcribed with Maxima first strand cDNA synthesis kit (Thermo Fisher). cDNA levels were analyzed by qRT-PCR amplification. Primers used were: *ACOX2* forward - AACCCAGGGGATCGAGTGT, *ACOX2* reverse – CGCAGCTCAGTGTTTGGGAT, β-actin forward – ATGCTCCCCGGGCTGTAT, β-actin reverse – CATAGGAGTCCTTCTGACC CATTC, *ATF4* forward, AGTGGCATCTGTATGAGCCCA, *ATF4* reverse – GCTCCTATT TGGAGAGCCCCT and *ATF5* forward – CTGGCTCCCTATGAGGTCCTTG, *ATF5* reverse – GAGCTGTGAAATCAACTCGCTCAG.

### siRNA mediated knockdown

The siRNAs used for the knockdown of ATF4, ATF5 and non-targeting (NC) were synthesized by DharmaconTM (Cambridge, UK). Initially the targeting efficiencies of siRNAs were tested at different concentrations and 20 nM concentration of each siRNA was chosen for actinonin treatment experiment. The inhibition efficiencies were detected by RT-qPCR. The siRNAs were transfected in human control fibroblasts (60−70% confluent) to a final concentration of 20 nM using Polyplus jetPRIME® transfection reagent (NY, USA). After 24 h of transfection, the cells were treated with actinonin (150 μM) for 24 h. The cells were then collected for RNA extraction, and the expression levels of *ATF4*, *ATF5* and *ACOX2* were quantified by RT-qPCR.

### Metabolite profiling

Mice were terminally anaesthetized with Pentobarbital (100 mg/kg) and blood was collected by heart puncture. The blood collection tubes were kept in ice for 15 min with serum separator and clot activator (MiniCollect Tube, Breiner-bion-one, 450533). The tubes were centrifuged at 5000RPM for 10 min and serum was transferred to a new tube and was kept at −80 °C until use. QF samples were collected from the same set of anaesthetized mice and snap frozen and kept at −80 °C until use. The metabolites were extracted from 20 mg mouse skeletal muscle (QF) tissue using 2 ml Precellys homogenization tube (Bertin Technologies,) with 2.8 mm ceramic (zirconium oxide) beads with 400 µl of cold extraction solvent (Acetonitrile:Methanol:H_2_O; 40:40:20). Subsequently, the samples were homogenized using tissue homogenizer (Bertin Technologies,) for 3 cycles (30 sec at 5500 rpm with 60 sec pause, at 4 °C). Furthermore, the homogenized samples were centrifuged at 14000 rpm at 4 °C for 5 min. For mouse serum, metabolites were extracted from 50 ul serum with 400 µL of cold extraction solvent (ACN:MeOH:H2O). After centrifugation the supernatant was loaded into Phree Phospholipid removal 96-well plate (8E-S133-TGB, Phenomenex) and passed through using robotic vacuum. The samples were analyzed with Thermo Vanquish UHPLC coupled with Q-Exactive Orbitrap quadrupole mass spectrometer equipped with a heated electrospray ionization (H-ESI) source probe (Thermo Fischer Scientific). A SeQuant ZIC-pHILIC (2.1 × 100 mm, 5 μm particle) column (Merck) was used for chromatographic separation. Gradient elution was carried out with a flow rate of 0.100 ml/min with using 20 mM ammonium hydrogen carbonate, adjusted to pH 9.4 with ammonium solution (25%) as mobile phase A and acetonitrile as mobile phase B. The gradient elution was initiated from 20% Mobile phase A and 80% of mobile phase B and maintained for 2 min., after that 20% Mobile phase A was gradually increased up to 80% for 17 min. Then Mobile phase A was decreased from 80% to 20% in 17.1 min and maintained up to 24 min. The column oven and auto-sampler temperatures were set to 40 ± 3 °C and 5 ± 3 °C, respectively. MS was equipped with a heated electrospray ionization (H-ESI) source using polarity switching and the following settings: resolution of 35,000, the spray voltages: 4250 V for positive and 3250 V for negative mode, the sheath gas: 25 arbitrary units (AU), and the auxiliary gas: 15 AU, sweep gas flow 0, Capillary temperature: 275 °C, S-lens RF level: 50.0. Instrument control was operated with the Xcalibur 4.1.31.9 software (Thermo Fischer Scientific).

In the data processing, the final peak integration was done with the TraceFinder 4.1 software (Thermo Fischer Scientific) using confirmed retention times of 462 in-house metabolites library developed using library kit MSMLS-1EA (Merck). For further data analysis, the peak area data was exported as excel file. The data quality was monitored throughout the run using pooled sample as Quality Control (QC) prepared by pooling 5 µL from each suspended samples and interspersed throughout the run as every 10th sample. After integration of QC serum data with TraceFinfer 4, the detected metabolites were checked for peak, % RSD were calculated, and acceptance limit was set to ≤ 30%. Blank samples for carryover were injected after every fifth randomized samples to monitor the metabolites carryover effect and calculated against the mean QC area and acceptance limit was set ≤ 20% for each metabolite. Background % noise was calculated with respect to first blank against the mean QC area and acceptance limit was set ≤ 20% for each metabolite.

### Metabolite data analysis

The data was analysed with Metaboanalyst 5.0, and the missing variables were imputated using KNNVAR. The values were normalised to the tissue weight, Log10 transformed and auto scaled. Following statistical analysis were performed: Univariate analysis methods: *t*-tests (FDR-adjusted p-value threshold 0.05, volcano plot (FC > 2, *t*-tests *p*-value < 0.05); Multivariate analysis methods: principal component analysis (PCA) and clustering analysis: heatmap (distance measure using euclidean, and clustering algorithm using ward.D). The differential expression was analyzed with lmFit using contrasts to compare knockout to wild type within each sample type. The differentially expressed metabolites (FDR < 0.01) were visualized with a volcano plot generated with ggplot2 (v.3.3.5). PCA was calculated with prcomp using the voom-normalized expression values. The enrichment analyses were performed for significantly altered metabolites (*P* < 0.05) using Metaboanalyst 5.0 (https://www.metaboanalyst.ca/) against KEGG database. To visualize pathway changes, bubble maps of enriched metabolites (significantly different *P* < 0.05) with mean log_2_ fold change (FC) were generated. Individual pathways or metabolites are plotted with mean log_2_FC and standard deviation.

### Statistics and reproducibility

The data are presented as mean ± SD, unless stated otherwise. The statistical analyses were performed with student’s *t*-test, and one-way or two-way ANOVA or as indicated in the figure legends. *P*-values less than 0.05 were considered significant. The significant differences between data or groups are indicated in the figures by *P < 0.05, **P < 0.01, ***P < 0.001. Statistics was performed with GraphPad Prism 9.2.0. The data analysis for RNA sequencing and metabolite profiling are explained in the respective method sections.

### Reporting summary

Further information on research design is available in the Nature Research Reporting Summary linked to this article.

## Supplementary information

Supplemental Material

Description of Additional Supplementary Files

Supplementary Data 1

Supplementary Data 2

Supplementary Data 3

Supplementary Data 4

Supplementary Data 5

Reporting Summary

## Data Availability

The results from the gene expression analysis together with the raw sequences have been deposited to GEO, accession GSE198540. The list of differentially expressed genes is in Supplementary Data 1 file. The pathway enrichments of the transcriptome data are in Supplementary Data 2 file. Transcriptome data for MitoCarta3.0 genes is in Supplementary Data 3 file. Metabolite profiling data from serum and muscle are in Supplementary Data 4 file. Source data underlying the graphs are provided in the Supplementary Data 5 file. Images of uncropped WB raw data are provided in Supplementary Figs. 5–7.
