# Supplementary material for: Inter-organellar and systemic responses to impaired mitochondrial matrix protein import in skeletal muscle
Source: Commun Biol. 2022 Oct 5;5:1060. doi: 10.1038/s42003-022-04034-z (PMC9534917; doi:10.1038/s42003-022-04034-z)
Supplement: Supplementary file 1 — Supplemental Material [file 42003_2022_4034_MOESM1_ESM.pdf]

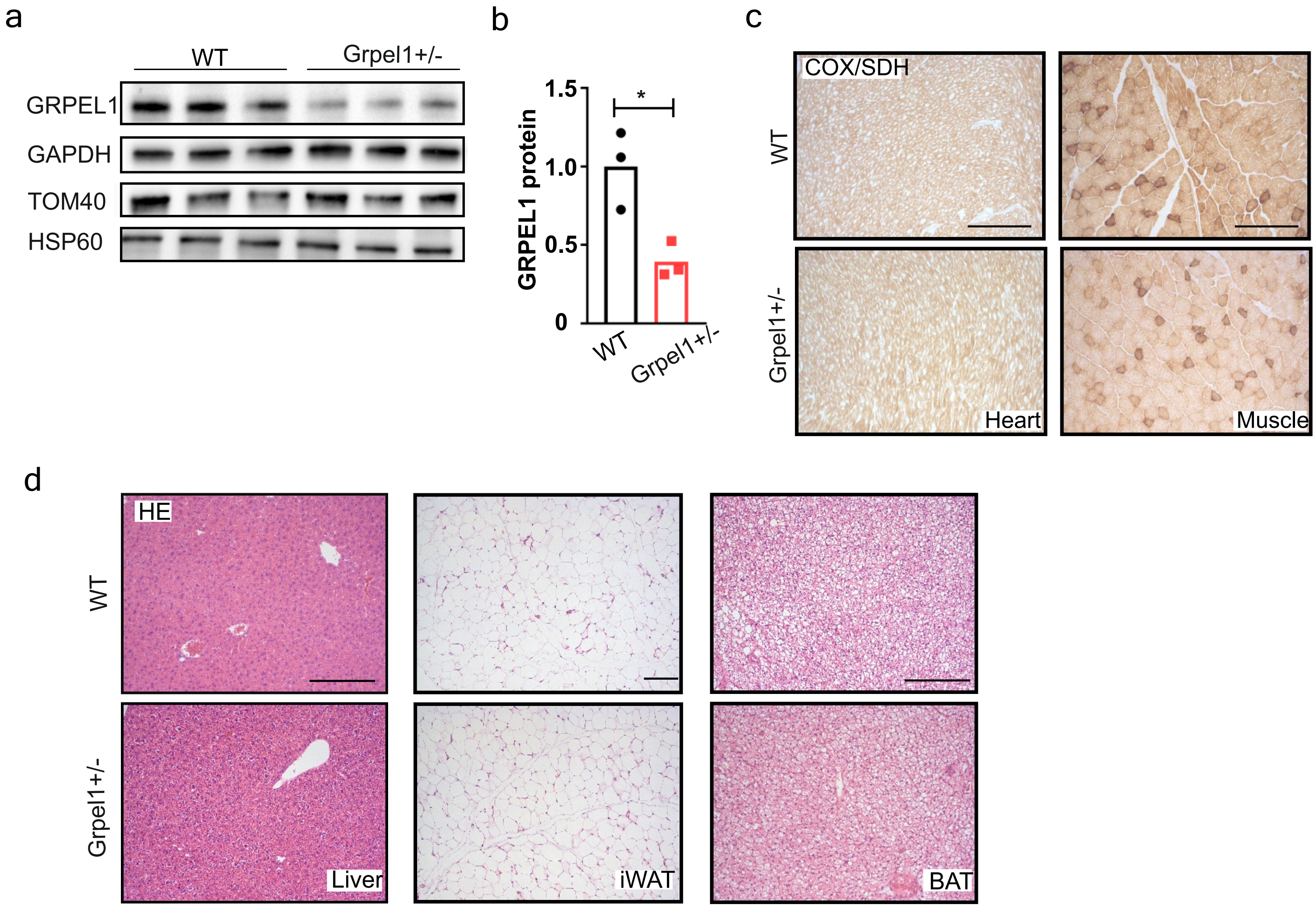

e

Grpel1+/- x Grpel1+/-	Genotypes					
	Expected			Observed		
	+/+	+/-	-/-	+/+	+/-	-/-
**Postnatal**	25%	50%	25%	36 (34.6%)	68 (65.3%)	0 (0%)
**Embryos E8.5**	25%	50%	25%	10 (41.6%)	14 (58.3%)	0 (0%)

Supplementary Fig. 1: Characterization of heterozygous Grpel1 knockout mice
a) Representative immunoblot showing reduced GRPEL1 in liver of heterozygous mice.
b) Quantification of GRPEL1 protein level in heterozygous mice.
c) Representative COX/SDH staining of the heart and skeletal muscle of 1-year-old wildtype and Grpel1+/- mice.
d) Representative Hematoxylin/Eosin staining of liver, inguinal white adipose tissue (iWAT) and brown adipose (BAT) of 1-year-old wild type and Grpel1+/- mice.
e) Punnett square showing the genotypes of the offspring from crossing of Grpel1+/- mice. No Grpel1-/- pups or E8.5 embryos were found.

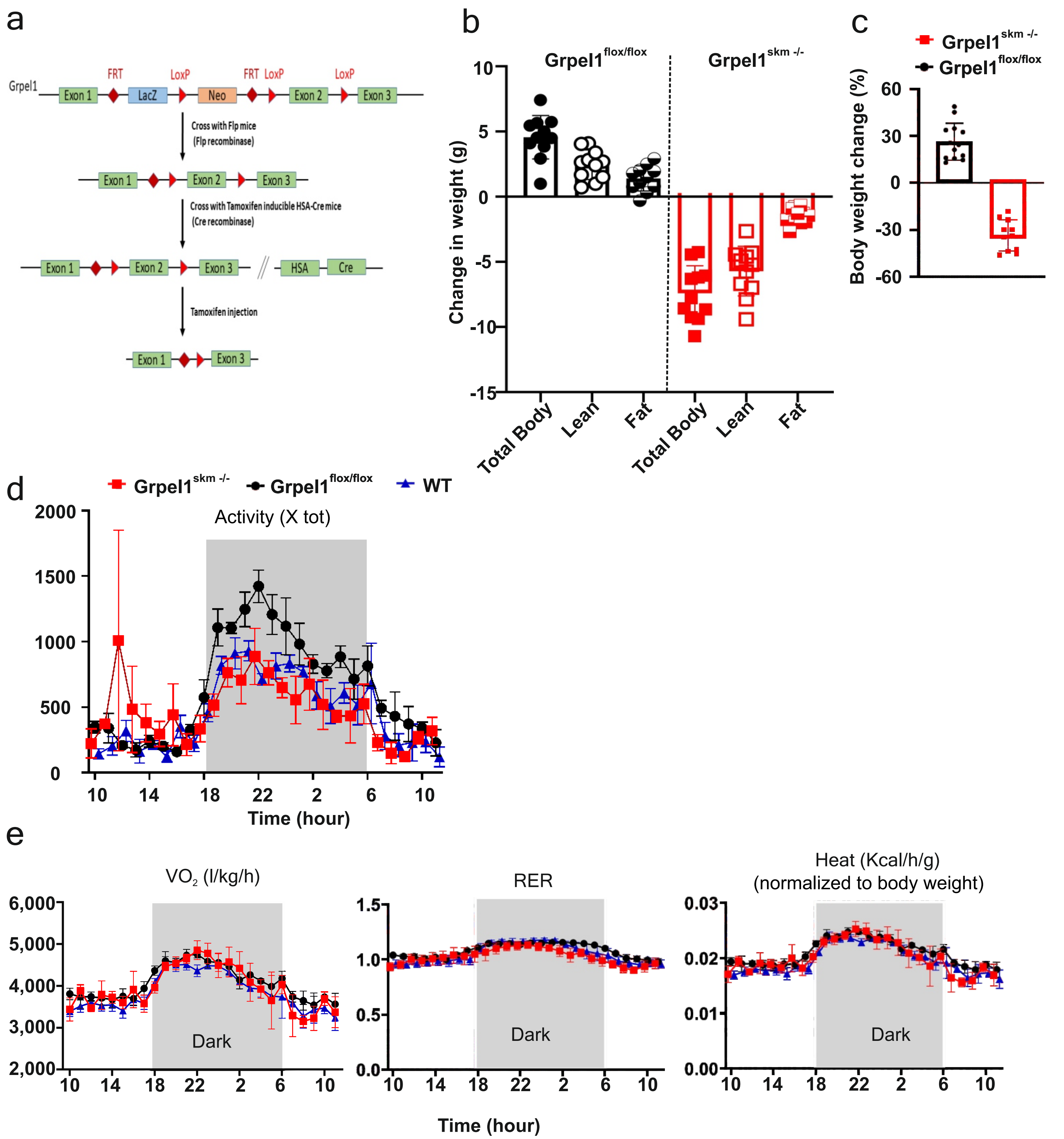

Supplementary Fig. 2: Body composition and indirect calorimetry studies of Grpel1$^{skm -/-}$ mice

a) Schematic representation of the *Grpel1$^{skm -/-}$* first allele.

b) Body composition analysis of *Grpel1$^{skm -/-}$* at the time of tamoxifen injections and at sacrification point showing the change in weight in total body weight and in lean and fat, in comparison to control mice (n=11 per genotype).

c) Percentage change in total body weight

d) Indirect calorimetry data showing the spontaneous activity of mice in the cage, two weeks after the tamoxifen injections (n=6 per genotype).

e) Oxygen consumption, respiratory exchange ratio (RER) and heat production in *Grpel1$^{skm -/-}$* compared to the controls, after two weeks of tamoxifen injections measured with indirect calorimetry.

Error bars represent the standard deviation.

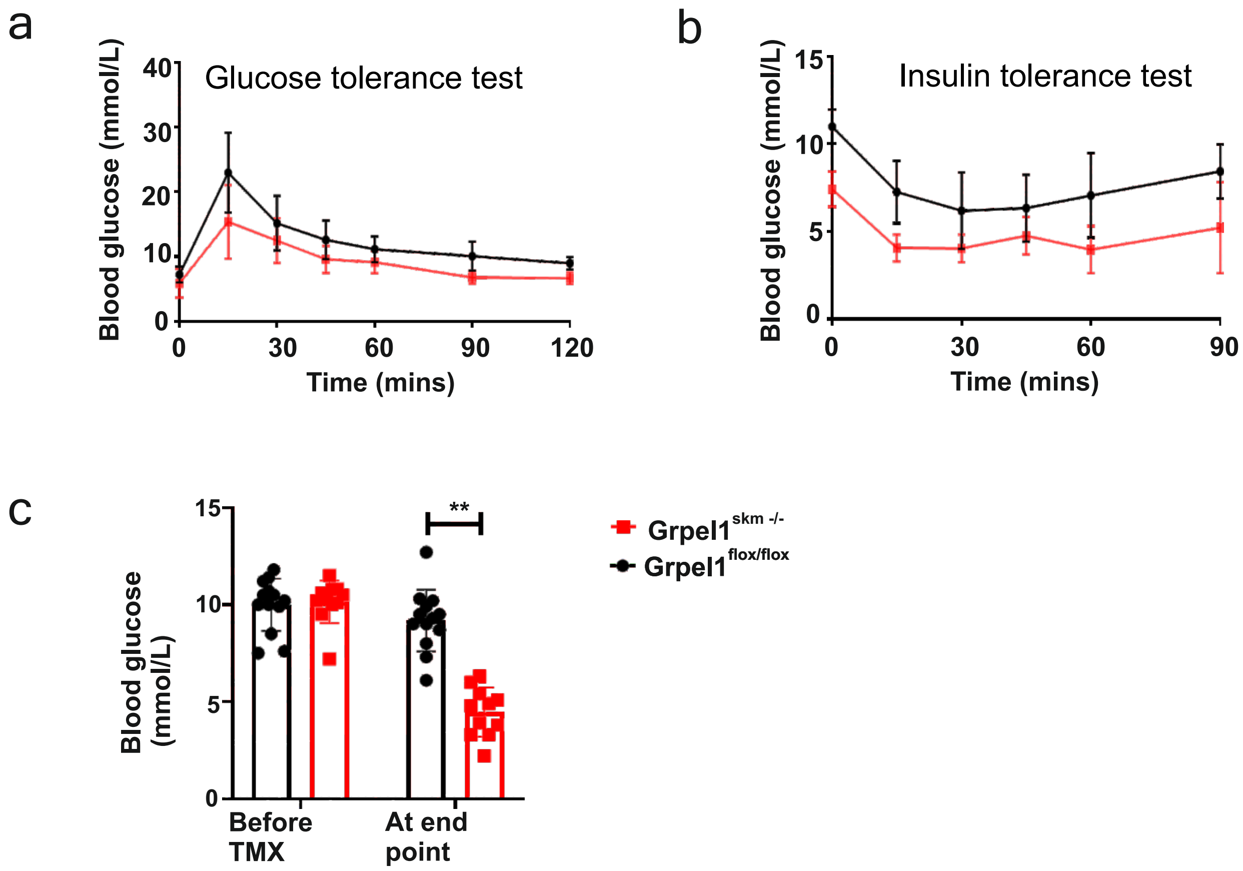

Supplementary Fig. 3: Blood glucose measurement and glucose and insulin tolerance test *Grpel1*$^{skm -/-}$ and control mice

a) Fasting (6 hour) glucose tolerance test in *Grpel1*$^{skm -/-}$ and control mice (n=5 per genotype).

b) Insulin tolerance test in *Grpel1*$^{skm -/-}$ and control mice (n=5 per genotype)

c) Non-fasted blood glucose level before tamoxifen injections and at sacrification (end) point (n=10-12 per genotype).

Error bars represent the standard deviation.

a

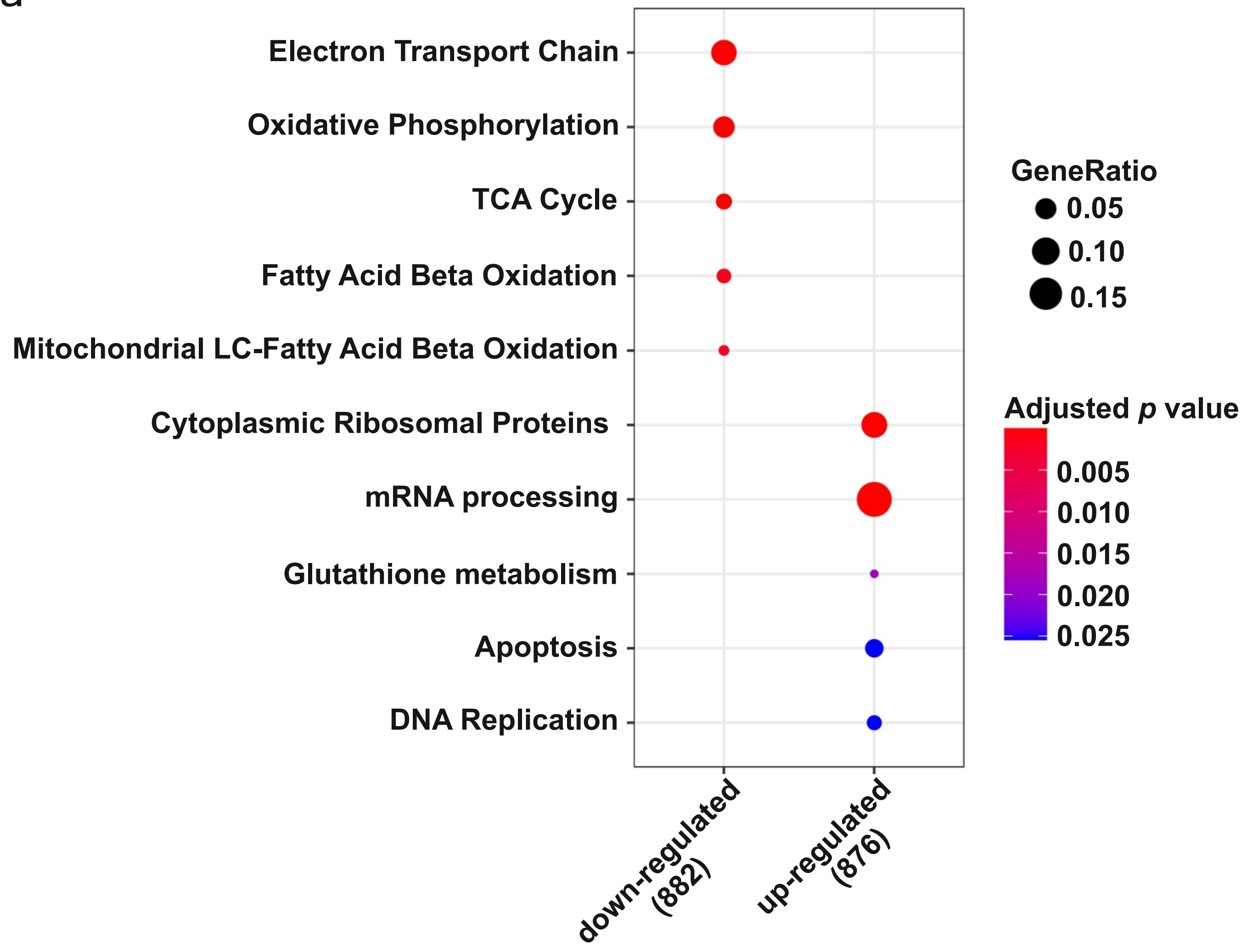

Supplementary Fig. 4: Pathway enrichment analysis

a) WikiPathways analysis of gene expression of *Grpel1*$^{skm\ -/-}$ mice in comparison to control mice, highlighting the downregulation of metabolic pathways and upregulation of transcripts of cytoplasmic protein synthesis. The full results of the enrichment analysis against the Reactome pathways, KEGG, Gene ontology and WikiPathways are in Supplementary Data 2.

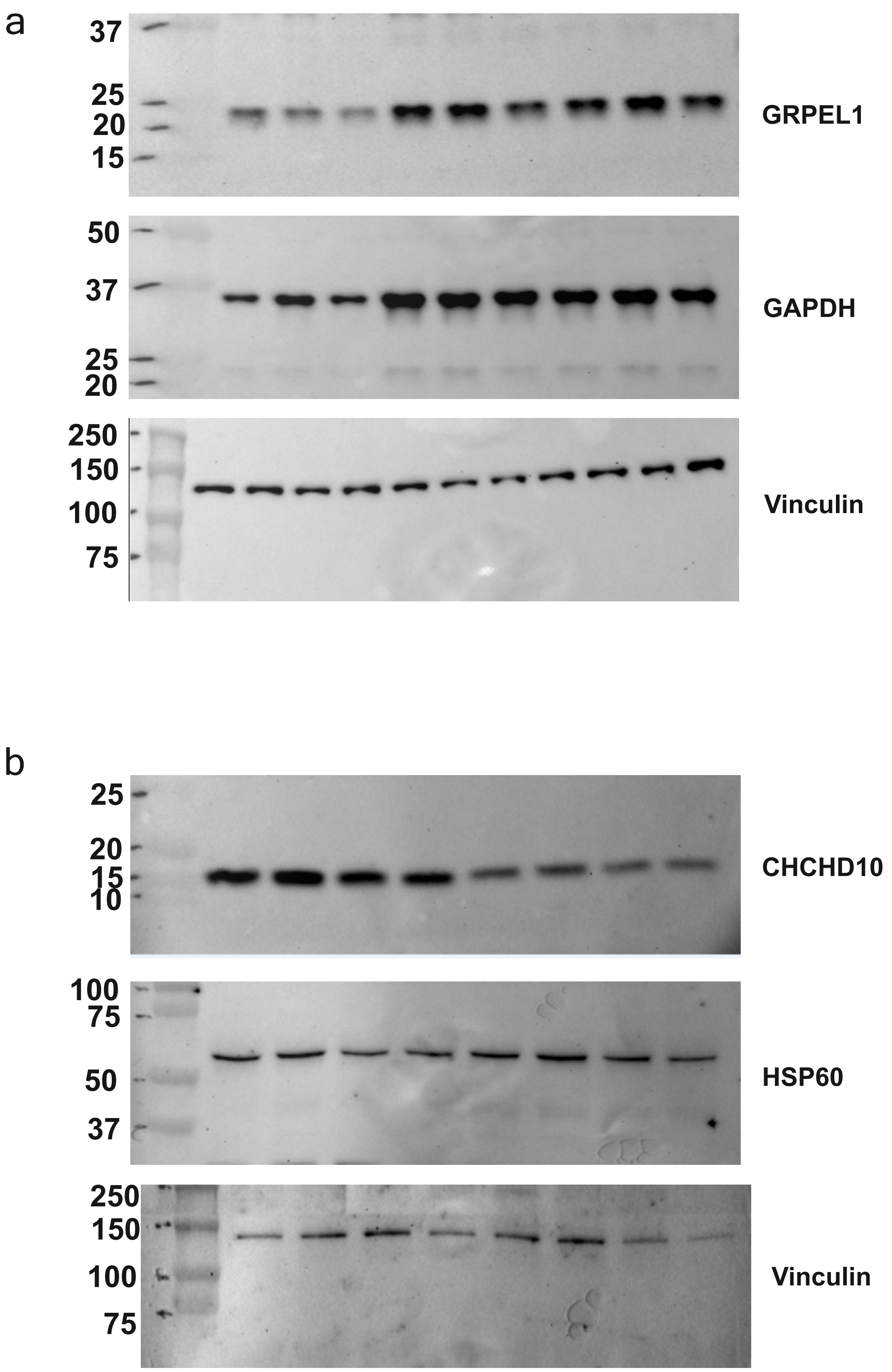

Supplementary Fig. 5: Original full blots of western blots
a) Original blots of images shown in Fig.1h.
b) Original blots of images shown in Fig. 2n.

a

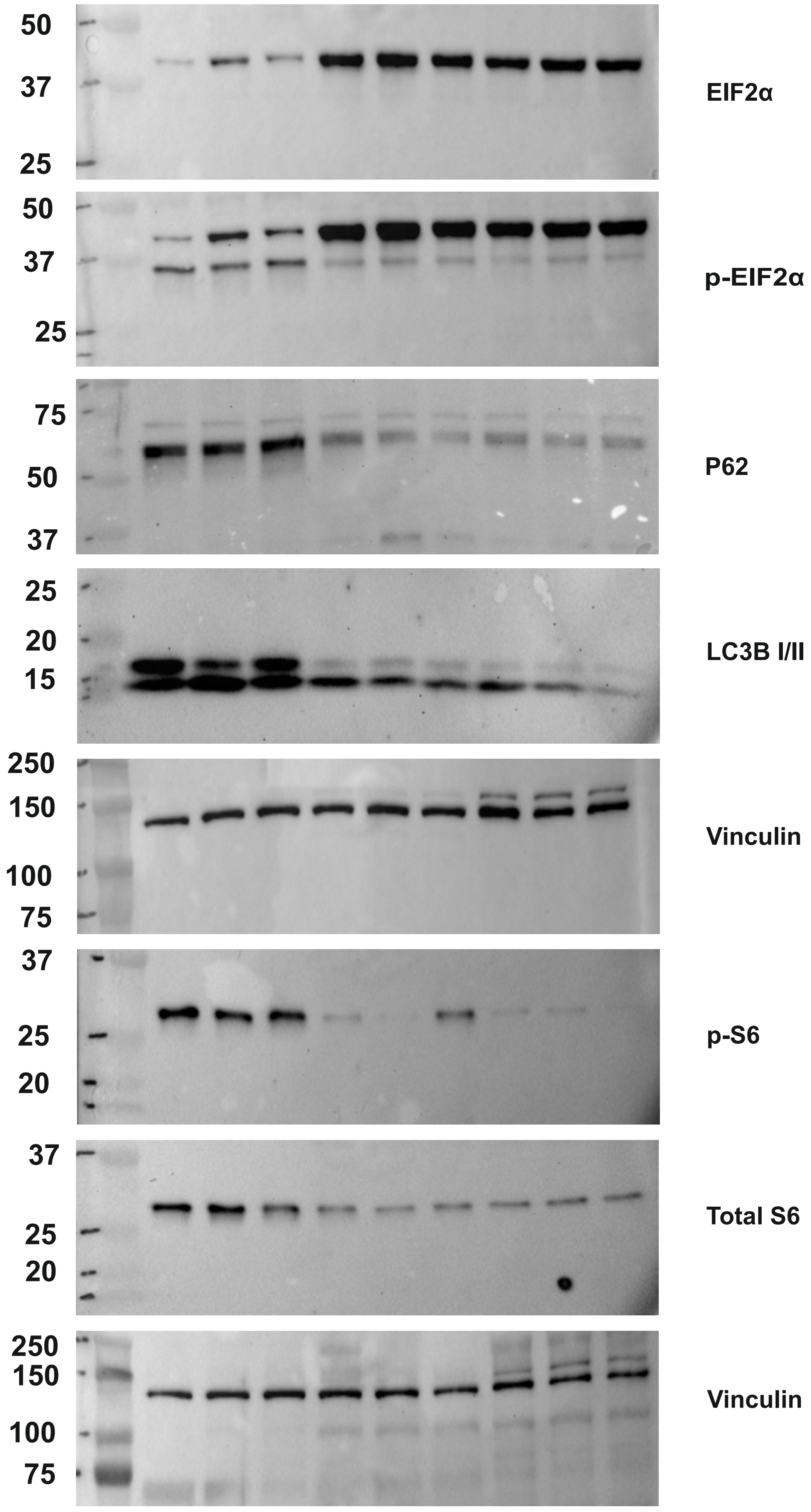

Supplementary Fig. 6: Original full blots of western blots
a) Original blots of images shown in Fig.3j.

a

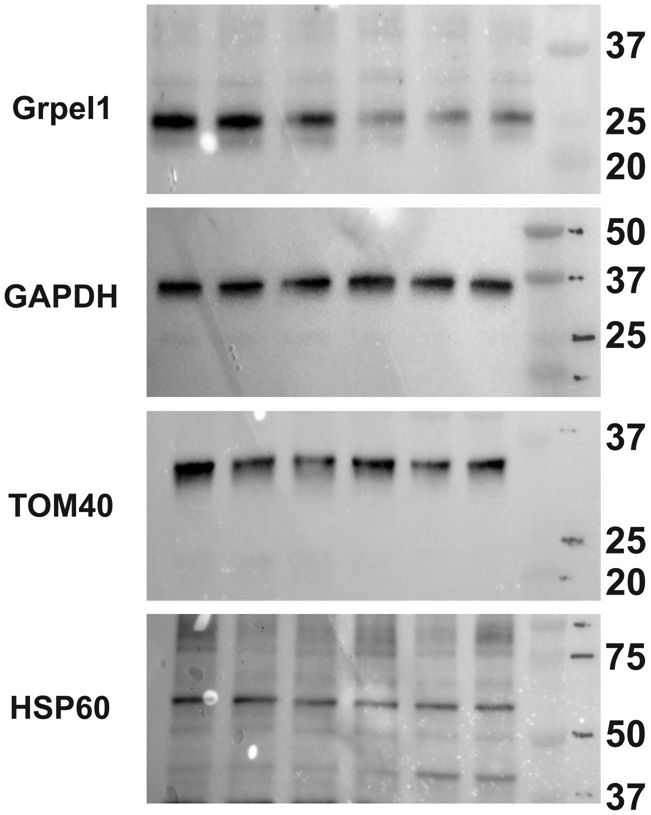

Supplementary Fig. 7: Original full blots of western blots

a) Original blots of images shown in Supplementary Fig1a.
