# Supplementary material for: Inter-organellar and systemic responses to impaired mitochondrial matrix protein import in skeletal muscle
Source: Commun Biol. 2022 Oct 5;5:1060. doi: 10.1038/s42003-022-04034-z (PMC9534917; doi:10.1038/s42003-022-04034-z)
Supplement: Supplementary file 2 — Description of Additional Supplementary Files [file 42003_2022_4034_MOESM2_ESM.pdf]

Description of Additional Supplementary Files

File name: Supplementary Data 1
Description: List of differentially expressed genes.

File name: Supplementary Data 2
Description: Pathway enrichments of the transcriptome data.

File name: Supplementary Data 3
Description: Transcriptome data for MitoCarta3.0 genes.

File name: Supplementary Data 4
Description: Metabolite profiling data from serum and muscle.

File name: Supplementary Data 5
Description: Numerical source data for graphs and charts.
